# Feasibility of bone-like MRI for acetabular morphology assessment in developmental dysplasia of the hip

**DOI:** 10.1186/s41747-026-00725-y

**Published:** 2026-04-27

**Authors:** Tetsuichi Hondera, Yasushi Yoshikawa, Daiho Kasahara, Shota Katsumata, Yoshiaki Hirai, Hisaya Sato

**Affiliations:** 1 Department of Radiological technology, Showa Medical University Hospital, Tokyo, Japan; 2 Showa Medical University Graduate School of Health Sciences, Yokohama, Japan; 3 Department of Orthopaedic Surgery, School of Medicine, Showa Medical University, Tokyo, Japan

**Keywords:** Bone and bones, Developmental dysplasia of the hip, Magnetic resonance imaging, Joint cartilage, Tomography (x‑ray computed)

## Abstract

**Objectives:**

To evaluate the clinical utility of “multi-echo recombined gradient echo” (MERGE)-based bone-like MRI for delineating acetabular osseous morphology in developmental dysplasia of the hip (DDH), and to determine whether this technique provides quantitative measurements with agreement to CT.

**Materials and methods:**

This retrospective study included 32 patients with DDH (2 men, 30 women; aged 33 ± 13 years, mean ± standard deviation) who underwent both hip MRI and CT between August 2020 and September 2023. One clinically affected hip per patient was analyzed. The MRI protocol included T1-weighted imaging, short tau inversion-recovery (STIR), and a MERGE sequence with a negative look-up table to enhance bone-like contrast. Joint space width (JSW) and center-edge (CE) angles were measured on all modalities.

**Results:**

Mean JSW was 4.2 ± 1.4 mm (T1-weighted), 3.0 ± 1.3 mm (STIR), 3.3 ± 0.9 mm (MERGE), and 3.4 ± 1.1 mm (CT), without a significant difference between MERGE and CT (*p* = 0.776). Mean CE angles were 29.7 ± 9.5°, 30.9 ± 9.9°, 31.4 ± 10.1°, and 31.4 ± 10.1°, respectively, without significant difference between MERGE and CT (*p* = 0.908).

**Conclusion:**

As an exploratory feasibility study, our findings suggest that MERGE-based bone-like MRI may allow reliable visualization of acetabular morphology and provide quantitative measurements with good agreement to CT. The ability to simultaneously evaluate osseous and soft-tissue structures supports its potential as a radiation-free and comprehensive imaging method for DDH, warranting further validation in larger cohorts.

**Relevance statement:**

MERGE-based bone-like MRI enables simultaneous visualization of the hip bone and soft tissues without radiation exposure. This method provides a clinically feasible, MRI-based alternative to CT for evaluating acetabular morphology in DDH.

**Key Points:**

- MERGE-based MR bone-like imaging visualized acetabular morphology and provided CE angle and joint space measurements comparable to CT.
- MERGE showed the closest agreement with CT for CE angle and joint space, outperforming T1WI and STIR in accuracy.
- This technique offers radiation-free, comprehensive hip evaluation, serving as a potential MRI-based alternative to CT in DDH assessment.

**Graphical Abstract:**

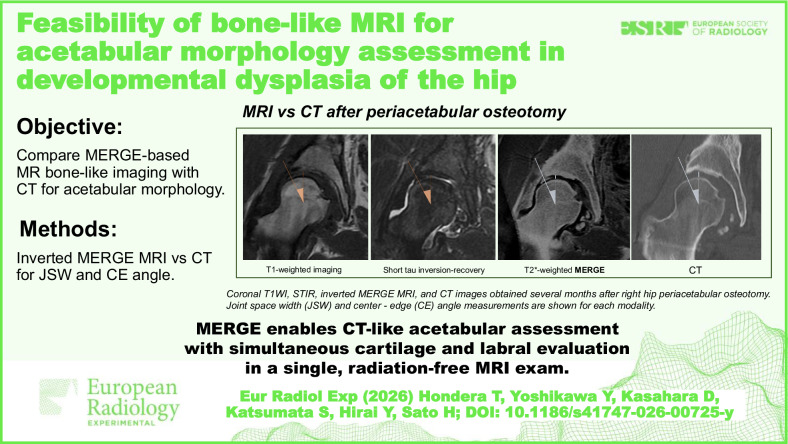

## Background

Developmental dysplasia of the hip (DDH) is a common disorder of the hip joint and represents a major cause of osteoarthritis in adulthood [1]. In DDH, insufficient acetabular coverage of the femoral head leads to uneven weight distribution across the articular surface, resulting in progressive wear of the articular cartilage and damage to the labrum. This structural degradation further impairs hip joint stability, ultimately progressing into osteoarthritis, characterized by pain and reduced range of motion.

Treatment options for DDH range from conservative management to surgical interventions. Among these, periacetabular osteotomy (PAO), a joint-preserving surgical procedure, is frequently performed in relatively young patients with early-stage osteoarthritis. These surgical techniques aim to improve the acetabular coverage by reorienting the acetabulum, thereby normalizing biomechanical load transmission and stabilizing the joint. Therefore, an accurate preoperative evaluation of acetabular coverage is essential for determining treatment suitability, planning surgical procedures, and establishing postoperative evaluation criteria [2].

Acetabular coverage is broadly classified into bony coverage and soft tissue coverage. Bony coverage reflects the osseous architecture and is typically assessed using plain radiographs or computed tomography (CT). Quantitative indices such as the center-edge (CE) angle and joint space width (JSW) are widely used for these evaluations [3]. Historically, JSW served as a surrogate for cartilage thickness in CT-based assessment, where cartilage is not visible. The CE angle is a well-established indicator of lateral acetabular coverage and plays a central role in preoperative planning. In contrast, JSW has historically been used as an indirect surrogate for cartilage thickness, particularly in CT-based assessments where cartilage cannot be visualized.

Using magnetic resonance imaging (MRI), direct visualization of cartilage is possible, enabling more precise assessment of joint integrity. With the increasing availability of MRI-based direct cartilage visualization, there is growing interest in whether JSW will remain clinically necessary or whether cartilage-based assessment may eventually replace it. These assessments, based on an anatomical understanding of bony structures, are consistently utilized from diagnosis to postoperative follow-up. Soft tissue coverage, mainly provided by the labrum and surrounding periarticular structures, plays a significant role in the dynamic stability of the hip joint. Evaluation of the femoral head, cartilage, and labrum is crucial in DDH, as these structures influence surgical decision-making and prognosis.

MRI provides unique diagnostic value by enabling direct assessment of the labrum, which is often affected earlier than bony morphology and has prognostic relevance. Consequently, a comprehensive evaluation of the hip joint currently requires a multimodal imaging approach combining CT and MRI [4]. Recent advances in MRI technology have led to the development of bone MRI techniques, which allow for a direct visualization of bone structures [5, 6]. Specialized sequences such as “ultrashort echo time” and “zero echo time” have been developed to visualize tissues with extremely short T2 or T2* relaxation times for cortical and cancellous bone, providing bone images comparable to those of CT [7–10].

The “multi-echo recombined gradient echo (MERGE) sequence, a T2*-weighted gradient-echo sequence commonly used in clinical practice, also provides excellent contrast between bone and soft tissues and has shown potential for visualizing bone structures. Although cortical bone typically appears as a low-signal-intensity area on conventional MERGE images due to its short T2* relaxation, applying a black-and-white inversion can visualize cortical bone with high signal intensity images, thereby enhancing the contrast with surrounding tissues. This post-processing technique visually emphasizes bone structures and produces CT-like images. Recent reports have indicated that such inverted MERGE images can yield visual representations comparable to those of dedicated bone MRI [11, 12]. However, quantitative validation and systematic assessment for clinical application remain limited.

In addition to acetabular morphology, femoral head morphology is clinically relevant for assessing femoroacetabular congruency, impingement risk, and early degenerative alterations in DDH. Although MERGE theoretically provides sufficient contrast for quantitative evaluation of femoral morphology, it remains uncertain whether MERGE can serve as a reliable alternative to CT for comprehensive assessment of both acetabular and femoral anatomy. This uncertainty has implications for workflow efficiency and cost-effectiveness, particularly if MERGE cannot ultimately eliminate the need for CT.

Therefore, the aim of this study was to conduct an exploratory feasibility assessment of whether gray-scale inverted MERGE images may be useful for quantitative evaluation of acetabular morphology in DDH. By comparing inverted MERGE images with CT measurements, this preliminary investigation seeks to determine whether MERGE-based bone visualization may have the potential to support a single-modality MRI workflow for preoperative assessment, although further validation is required. Importantly, this study focuses on evaluating bone structures relevant to joint-preserving treatments such as conservative management and PAO, rather than preoperative assessments for total hip arthroplasty or arthroscopic surgery.

## Methods

### Study design and participants

This study was designed as a retrospective, single-center, exploratory feasibility investigation to assess whether the MERGE sequence obtained from hip MRI could be utilized for bone structure evaluations comparable to CT, in the context of MR bone-like imaging. As this investigation was designed as an exploratory feasibility study, no a priori power calculation was performed. This retrospective study included 32 patients with DDH, in whom both hip MRI and CT examinations were performed between August 2020 and September 2023. Only one hip per patient was analyzed. Specifically, the affected hip was selected for analysis in all cases. Bilateral hips from the same patient were not included, thereby ensuring independence of observations. All patients had either a confirmed clinical diagnosis of DDH or were referred due to suspected dysplasia based on physical examination or preliminary radiographic findings. Furthermore, some cases included postoperative patients who underwent imaging for follow-up evaluation of acetabular morphology, and in several cases, the final confirmation of DDH was made after the MRI and CT examinations. Inclusion criteria were: patients who underwent both hip MRI and CT on the same day for diagnostic or preoperative evaluation of acetabular morphology. Exclusion criteria were: (1) severe femoral head displacement preventing reliable measurement of JSW and CE angle; (2) metal implants that could generate artifacts; and (3) poor image quality due to motion or acquisition failure. None of the included cases demonstrated metal-induced artifacts. The interval between MRI and CT acquisition was 0 days, as all paired examinations were performed during the same hospital visit. This study was approved by the Ethics Committee on Research Involving Human Subjects at the institution the authors belong to (approval number: 22-139-B).

In addition, because DDH can present bilaterally, the potential clustering effect of bilateral hip data was considered at the study design stage. Therefore, the primary analysis was restricted a priori to one hip per patient by selecting the clinically affected side only. As a sensitivity analysis, key agreement and reliability metrics (intraclass correlation coefficient [ICC] and Bland–Altman parameters) were re-evaluated at the patient level, and the consistency of the results was confirmed. Because this study was designed as an exploratory feasibility investigation, no a priori sample size or power calculation was performed. Instead, the analysis focused on agreement- and precision-based metrics, including ICC, standard error of measurement (SEM), 95% minimal detectable change (MDC95), and Bland–Altman limits of agreement.

### MRI protocol

All MRI examinations were performed using a 1.5-T MRI scanner (Signa HDxt 1.5 T, GE Healthcare). The imaging protocol included coronal T1-weighted imaging (T1WI), coronal short tau inversion-recovery (STIR), and coronal MERGE sequences. Both standard MERGE and grayscale-inverted MERGE images were acquired; A look-up table was applied post-acquisition to generate the inverted images. The MERGE sequence, a T2*-weighted sequence developed by GE Healthcare [13], was specifically investigated as a potential bone-like MRI technique in this study. Similar sequences from other manufacturers include “multi-echo data image combination”‒MEDIC from Siemens Healthineers and “merged fast field echo”‒M-FFE from Philips Healthcare. MERGE can be acquired in either two-dimensional (2D) or three-dimensional (3D) modes and is characterized by providing high contrast between bone, cerebrospinal fluid, and soft tissues. It has been reported for its utility in evaluating cartilage thickness in early diagnosis of osteoarthritis in musculoskeletal imaging and for detecting cervical spinal lesions of multiple sclerosis in neuroimaging.

During the study period, only the 1.5-T system was available, and the scanner was later replaced by a 3-T MAGNETOM Vida (Siemens Healthineers), making additional imaging with the previous system impossible. Moreover, the 1.5-T system did not support 3D MERGE acquisitions. Importantly, as noted in the Discussion, clinical requests from physicians prioritized 2D imaging rather than 3D acquisitions, further justifying the use of 2D MERGE for this study. For this study, the 2D acquisition mode was selected for the MERGE sequence to ensure consistent slice alignment with the other routinely acquired 2D sequences (T1WI and STIR). This consistency is crucial for clinicians to effectively compare and integrate information from all sequences during routine diagnostic evaluations.

This approach reflects routine clinical practice and aligns with the imaging strategy used by orthopedic surgeons when assessing acetabular morphology. Detailed acquisition parameters for all MRI sequences are presented in Table 1.

**Table 1:** Hip MRI protocol

Sequence	T1WI	STIR	MERGE
Plane	Coronal (2D)	Coronal (2D)	Coronal (2D)
TR (ms)	540	4,520	515
TE (ms)	6.6	52.9	14.1
Matrix size (frequency × phase)	320 × 224	288 × 224	384 × 256
FOV (mm)	340 × 340	340 × 340	340 × 340
FA (degree)	90	-	20
ETL	3.0	11	11
Slice thickness (mm)	4.0	4.0	4.0
rBW (kHz)	50	41.7	83.3
NEX	2	1	1
TI (ms)	-	150	-
Coil	12-channel body array	12-channel body array	12-channel body array

*ETL* Echo train length, *FA* Flip angle, *FOV* Field of view, *MERGE* Multi-echo recombined gradient echo, *NEX* Number of excitations, *rBW* Receiver bandwidth, *STIR* Short tau inversion-recovery, *T1-WI* T1-weighted imaging, *TE* Echo time, *TI* Inversion time, *TR* Repetition time

### CT protocol

Examinations were performed using multidetector scanners (SOMATOM Definition AS + , SOMATOM X.cite, and SOMATOM Force; Siemens Healthineers). All scans were acquired in the supine position according to the routine clinical hip CT protocol at our institution. Acquisition parameters were standardized as follows: tube voltage of 120 kV, tube current of 250 mA, and axial slice thickness of 0.75 mm with a slice increment of 0.7 mm.

Images were reconstructed using a standard bone reconstruction kernel (Br64). Multiplanar reformatted images in the coronal plane were generated with a slice thickness of 2 mm and an interslice gap of 2 mm for quantitative measurements. No additional geometric correction, pelvic tilt adjustment, or image registration was applied, reflecting routine clinical measurement conditions, as summarized in Table 2.

**Table 2:** CT acquisition and reconstruction parameters

Parameter	Setting
CT system	SOMATOM Definition AS+, SOMATOM X.cite, SOMATOM Force (Siemens Healthineers)
Tube voltage (kV)	120
Tube current (mA)	250
Slice thickness (mm)	0.75
Slice increment (mm)	0.7
Reconstruction kernel	Bone kernel (Br64)
MPR plane	Coronal
MPR slice thickness (mm)	2.0
MPR slice increment (mm)	2.0
Patient position	Supine
Geometric correction	None
Pelvic alignment correction	None

*CT* Computed tomography, *MPR* Multiplanar reconstruction

All CT images were exported in Digital Imaging and Communications in Medicine (DICOM) format and reviewed using the same clinical image viewer as MRI (i-Rad QA; Fujifilm Medical Systems).

### Quantitative image analysis

**Fig. 1:**
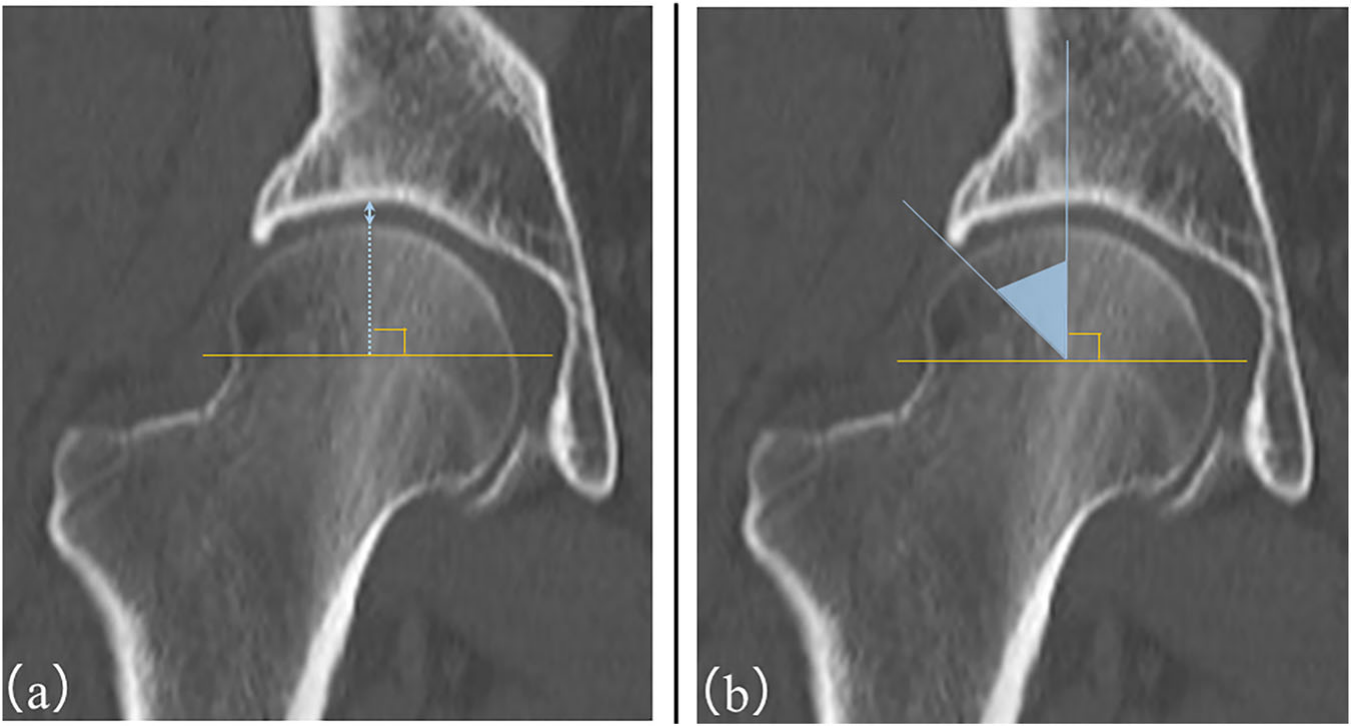
Measurement methods of JSW and CE angle. **a** JSW was measured at the 12 o’clock position from the center of the femoral head on coronal images at the level of the femoral head center. **b** The CE angle was measured as the angle between a vertical line passing through the center of the femoral head and a line connecting the center of the femoral head to the lateral edge of the acetabulum, on the same level

Quantitative image analysis was performed using T1WI, STIR, MERGE sequences, and coronal CT images. The evaluated parameters were JSW and the CE angle (Fig. 1). The detailed measurement procedures are described below. In this feasibility study, JSW was measured only along the 0° direction on coronal images, which reflects the most commonly assessed weight-bearing region in routine clinical practice. Similarly, acetabular morphology was evaluated using the CE angle as a representative and widely accepted parameter for lateral acetabular coverage.

The present study did not include multi-directional JSW assessment, pelvic tilt or rotation correction, or image-to-image registration between MRI and CT, as the primary aim was to evaluate whether MERGE-based MR bone-like imaging could provide clinically comparable measurements under standard diagnostic conditions. Accordingly, the present analysis was intended to reflect routine clinical measurement conditions rather than geometrically standardized or research-oriented image alignment, and the results should be interpreted within this pragmatic clinical context.

### Measurement of JSW

JSW was measured on coronal images from T1WI, STIR, MERGE, and CT displayed on a medical image viewing monitor. Measurements were standardized by selecting the slice containing the center of the femoral head of the affected hip. The JSW was measured along a line extending from the center of the femoral head in the 0° direction.

### Measurement of CE angles

The CE angle was measured using the same coronal images as for JSW (T1WI, STIR, MERGE, and CT). The CE angle was defined as the angle formed by a vertical line passing through the center of the femoral head and a line connecting the center of the femoral head to the lateral acetabular edge.

### Evaluation criteria and measurement procedure

All measurements were independently performed by two radiological technologists with 9 and 13 years of clinical experience, respectively. The final measurement value for each parameter was determined by averaging the measurements from the two observers. The measurements obtained from the MRI sequences were compared with those from CT images to evaluate which MRI sequence provided values most consistent with CT. Importantly, only the grayscale-inverted MERGE images were used for quantitative analysis, as they provided sufficient bone-like contrast for comparison with CT. The original (non-inverted) MERGE images were not included in the evaluation.

### Interobserver reliability

To evaluate the reliability of measurements obtained by the two radiological technologists, ICC, SEM, and MDC95 were calculated for the JSW and CE angle. A two-way random-effects model with absolute agreement for single measurements—ICC(2,1)—was selected, as it is the most appropriate model when assessors are considered interchangeable and absolute agreement is required.

In addition, Bland–Altman analysis was performed for each modality to visually and quantitatively assess systematic bias (mean difference) and the 95% limits of agreement. After confirming good-to-excellent inter-observer reliability (ICC ≥ 0.75), the final measurement value for each parameter was defined as the mean of the two observers’ measurements (Table 4).

ICC values were interpreted as follows: less than 0.5, poor; 0.5 to 0.75, moderate; 0.75 to 0.9, good; and greater than 0.9, excellent [14]. SEM was calculated as the square root of the mean square error (MS_Error derived from the two-way random-effects ANOVA corresponding to ICC(2,1)):

$${{{\rm{SEM}}}}=\surd ({{{\rm{MS}}}}\_{{{\rm{Error}}}})$$

MDC95, defined as the minimum amount of change that can be interpreted as a true change rather than measurement error, was derived using the formula:

$${{{\rm{MDC}}}}95=1.96\times {{{\rm{SEM}}}}\times \surd 2.$$

Because the primary analysis was conducted using one hip per patient, the assumption of independence was maintained for the main results. Nevertheless, as a sensitivity analysis, agreement and reliability metrics were re-evaluated at the patient level to confirm that the conclusions were not materially altered by within-patient clustering.

### Statistical analysis

The associations between the measured values of the JSW and CE angle obtained from T1WI, STIR, MERGE, and CT images were evaluated. Statistical analysis was performed using JMP Pro version 17 (SAS Institute Inc.). Data are presented as mean ± standard deviation, unless otherwise specified. The Wilcoxon signed-rank test was used to assess differences between groups. A p-value of < 0.01 was considered statistically significant. Interobserver reliability for JSW and CE angle measurements was assessed using the ICC. Agreement between MERGE and CT measurements for JSW and CE angle was further assessed using Bland–Altman analysis. The mean difference and 95% limits of agreement (mean ± 1.96 standard deviation) were calculated to evaluate systematic and proportional bias between modalities.

No formal equivalence testing (*e.g*., two one-sided tests) was performed; therefore, results are described in terms of agreement, bias, and measurement consistency rather than statistical equivalence.

## Results

### Participants

A total of 32 consecutive patients (2 males and 30 females; age 33 ± 13 years, mean ± standard deviation) met the inclusion criteria and were included in the analysis. Only one hip per patient was analyzed; therefore, a total of 32 hips from 32 patients were evaluated (Table 3). As specified in the “Methods” section, cases with severe superolateral displacement of the femoral head, which prevented reliable measurement of JSW CE angle, were excluded. All included cases satisfied the predefined inclusion criteria, and no excluded cases demonstrated metal-induced artifacts or incomplete imaging datasets.

**Table 3:** Patients characteristics

Age (years)	33.1 ± 13.1
Sex (men)	2
Sex (women)	16
Sex (% women)	89
Affected side (% right)	81
Height (m)	1.57 ± 0.05
Weight (kg)	62.0 ± 12.1

Data are presented as %, mean ± standard deviation, or frequencies

### JSW

**Fig. 2:**
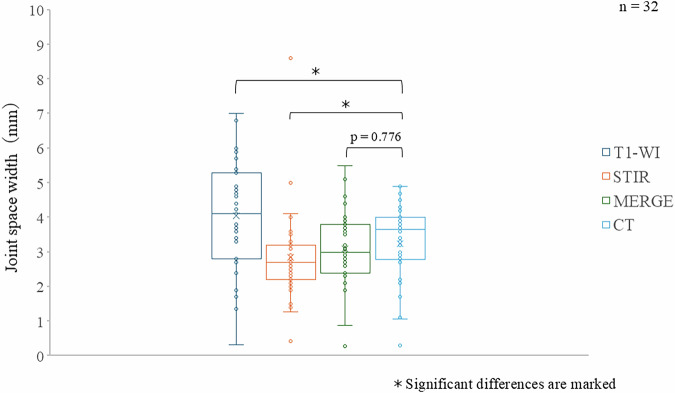
Results of JSW measurements. In the 32 hips analyzed in this study, the mean JSW measured on MRI was 4.2 ± 1.4 mm on T1-weighted images (T1WI), 3.0 ± 1.3 mm on STIR, and 3.3 ± 0.9 mm on MERGE images. On CT images, the mean JSW was 3.4 ± 1.1 mm. CT, Computed tomography; MERGE, Multi-echo recombined gradient echo; STIR, Short tau inversion-recovery; T1-WI, T1-weighted imaging

**Fig. 3:**
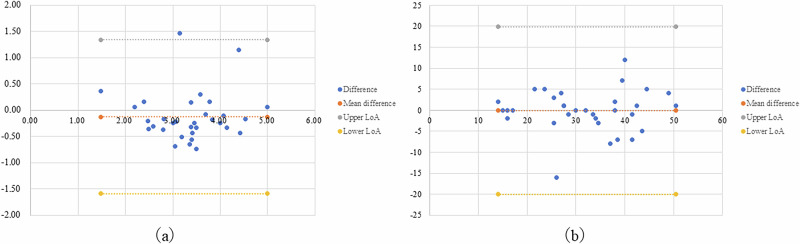
Bland–Altman analysis comparing MERGE MRI and CT measurements. **a** Bland–Altman plot showing agreement between MERGE MRI and CT measurements for JSW. The solid horizontal line represents the mean difference (-0.25 mm), and the dashed lines indicate the 95% limits of agreement (-2.04 to 1.54 mm). No proportional bias was observed across the measurement range. **b** Bland–Altman plot showing agreement between MERGE MRI and CT measurements for the CE angle. The solid horizontal line represents the mean difference (-0.09°), and the dashed lines indicate the 95% limits of agreement (-9.85° to 9.66°). No systematic or proportional bias was observed. CE, Center-edge; CT, computed tomography; MERGE, Multi-echo recombined gradient echo

For the 32 hips included in this study, the measured JSW on the MRI images was 4.2 ± 1.4 mm for T1WI, 3.0 ± 1.3 mm for STIR, and 3.3 ± 0.9 mm for MERGE. Here, the average JSW measured on the CT images was 3.4 ± 1.1 mm. Among the MRI sequences, MERGE measurements showed the closest approximation to the CT measurements, with a mean difference (error) of 0.1 ± 0.2 mm. Statistical analysis showed significant differences in JSW between T1WI and CT (*p* < 0.01) and/or between STIR and CT (*p* < 0.01). However, there were no significant differences between MERGE and CT (*p* = 0.776) with JSW (Fig. 2). Bland–Altman analysis demonstrated moderate agreement between MERGE and CT measurements for JSW, with a small mean difference of -0.25 mm and 95% limits of agreement ranging from -2.04 to 1.54 mm. Although the limits of agreement were relatively wide, these differences largely reflect modality-related differences in cartilage depiction rather than random measurement error (Fig. 3a).

### CE angle

**Fig. 4:**
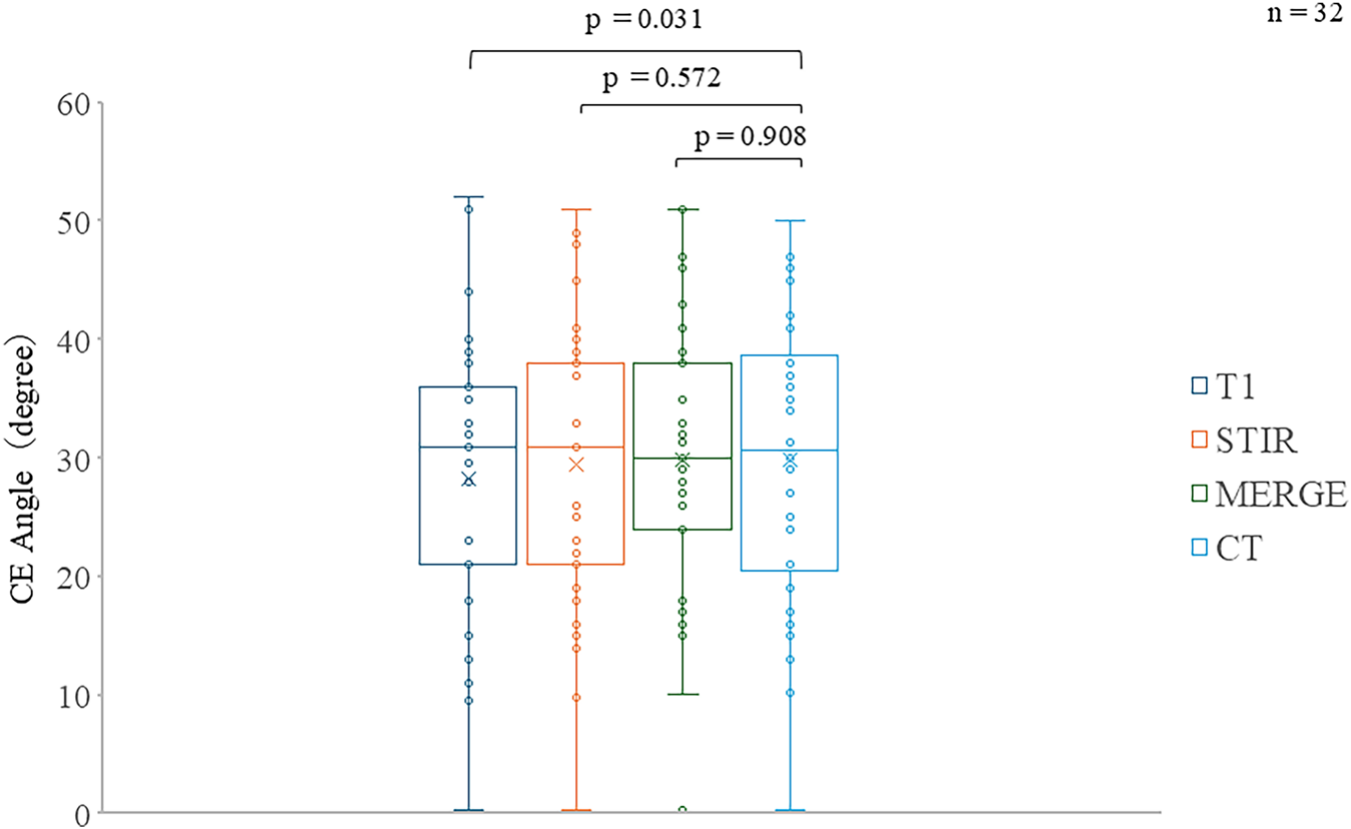
Results of CE angle measurements. In the 32 hips analyzed, the mean CE angle measured on MRI was 29.7 ± 9.5° on T1WI, 30.9 ± 9.9° on STIR, and 31.4 ± 10.1° on MERGE images. On CT, the mean CE angle was 31.4 ± 10.1°. CT, Computed tomography; MERGE, Multi-echo recombined gradient echo; STIR, Short tau inversion-recovery; T1-WI, T1-weighted imaging

For the 32 hips included in this study, the measured CE angles on the MRI images were 29.7 ± 9.5 degrees for T1WI, 30.9 ± 9.9 degrees for STIR, and 31.4 ± 10.1 degrees for MERGE. The CE angle measured on CT images was 31.4 ± 10.1 degrees, indicating that MERGE measurements showed a negligible mean difference and good agreement with CT measurements. Statistical analysis showed no significant differences in the CE angle between all of the MRI sequences and CT images (T1WI *versus* CT, *p* = 0.031; STIR *versus* CT, *p* = 0.572; MERGE *versus* CT, *p* = 0.908) (Fig. 4). Bland–Altman analysis further confirmed good agreement between MERGE and CT for CE angle measurements, with a negligible mean difference of -0.09° and 95% limits of agreement ranging from -9.85° to 9.66°. No systematic or proportional bias was identified, supporting the consistency of CE angle measurements between MERGE and CT (Fig. 3b).

### Sensitivity analysis

When the analyses were repeated at the patient level using one hip per patient, the direction and magnitude of the mean differences, ICC values, and limits of agreement between MERGE and CT remained consistent with the primary analysis. These findings indicate that the overall conclusions were robust and not materially influenced by within-patient clustering.

### Inter-observer reliability

Inter-observer reliability was evaluated for measurements of JSW and the CE angle across all imaging modalities. The results of ICC(2,1), SEM, and MDC95 are summarized in Table 4. The overall ICC for JSW measurements across all modalities was 0.92 (95% confidence interval: 0.88–0.95), and that for CE angle measurements was 0.94 (95% confidence interval: 0.91–0.97). A modality-specific analysis revealed high measurement reliability for all sequences except JSW measured on T1WI (ICC = 0.564). MERGE demonstrated excellent reliability for CE angle (ICC = 0.970) and good reliability for JSW (ICC = 0.886), with SEM and MDC95 values comparable to those of CT. These findings confirm that MERGE provides robust measurement performance with low measurement error. Although the limits of agreement for JSW were relatively wide, differences within approximately ±2 mm should be interpreted with consideration of the overall clinical context. These findings support the feasibility of MERGE for pragmatic clinical assessment rather than strict interchangeability with CT. To confirm the robustness of our findings, a sensitivity analysis was performed, including all available hips (both sides). The agreement between MERGE and CT remained high for both the JSW (ICC = 0.781, 95% CI: 0.655–0.865) and CE angle (ICC = 0.916, 95% CI: 0.865–0.948), consistent with the results of the primary one-hip-per-patient analysis.

**Table 4:** Interobserver reliability of JSW and CE angle measurements across MRI sequences and CT

Parameter	Modality	ICC (2, 1)	95% CI	SEM	MDC95
JSW (mm)	T1	0.564	(0.052, 0.849)	0.523 mm	1.45 mm
	STIR	0.951	(0.844, 0.988)	0.133 mm	0.37 mm
	MERGE	0.886	(0.697, 0.969)	0.095 mm	0.26 mm
	CT	0.955	(0.871, 0.990)	0.049 mm	0.14 mm
CE angle (°)	T1	0.959	(0.875, 0.991)	2.590°	7.18°
	STIR	0.963	(0.887, 0.992)	2.450°	6.80°
	MERGE	0.970	(0.908, 0.994)	2.270°	6.30°
	CT	0.957	(0.871, 0.991)	2.500°	6.94°

*CE* Center-edge, *CI* confidence interval, *CT* Computed tomography, *ICC* Intraclass correlation coefficient, *JSW* Joint space width, *MDC95* Minimal detectable change at 95% confidence level, *MERGE* Multi-echo recombined gradient echo, *SEM* Standard error of measurement, *STIR* Short tau inversion-recovery, *T1WI* T1-weighted imaging

## Discussion

### Technical basis of MERGE for bone-like MRI

This study investigated the utility of the MERGE sequence in hip MRI for visualizing bone structures akin to CT. MERGE is a T2*-weighted sequence that generates images with combined T1 and T2* contrast through the reconstruction of signals acquired at multiple echo times, enabling high-contrast visualization of both articular cartilage and bone [15]. A distinctive technical aspect of this study was the post-processing application of grayscale (black-and-white) inversion to MERGE images, transforming the inherently low signal intensity of cortical bone—typically observed due to its short T2* relaxation—into a region of high signal intensity. This inversion effectively enhances the contrast of bone structures against surrounding soft tissues, thus mimicking CT images. Importantly, this inversion processing was performed simply and reproducibly by applying a negative “look-up table” to DICOM images using the Fujifilm Medical image viewer i-Rad QA. The feasibility of performing this visualization technique on a general-purpose clinical viewer, without specialized analysis software or dedicated hardware, holds significant implications for inter-facility reproducibility and broader clinical adoption.

### Quantitative evaluation and reliability

Agreement and reproducibility were assessed using intraclass correlation coefficients (ICC) and Bland–Altman analysis, thereby avoiding dependence on non-significant *p*-values. Quantitative evaluation focused on two key parameters of bone morphology, and inter-observer reliability for both JSW and CE angle was excellent (ICC ≥ 0.886 for MERGE) (Table 4). These results confirm that the measurement procedures were highly reproducible and that agreement was strong across modalities. Collectively, these findings support the robustness of MERGE-based MR bone-like imaging for quantitative assessment of hip morphology. In addition, Bland–Altman analysis demonstrated small mean differences between MERGE and CT for both JSW and CE angle measurements, with narrow limits of agreement and no evidence of systematic or proportional bias. These findings further support the measurement-level agreement between MERGE and CT beyond correlation-based metrics. Importantly, the width of the limits of agreement for JSW should be interpreted in the clinical context of DDH, as JSW represents a small absolute distance and is sensitive to differences in cartilage depiction between imaging modalities. Therefore, the observed limits likely reflect biological and modality-related differences rather than clinically unacceptable measurement error.

### Interpretation of JSW measurements

However, MERGE tended to slightly underestimate JSW compared with CT, which is explained by its visualization of both cartilage and bone. Unlike CT, MERGE allows direct assessment of cartilage; therefore, JSW measured on MERGE may reflect cartilage-included distances rather than purely subchondral bone-to-bone distances. This highlights the importance of considering cartilage depiction when interpreting JSW in MRI. CT does not depict cartilage and thus measures the distance between subchondral bone surfaces (from the femoral head cortex to the acetabular cortex). Accordingly, differences between MERGE- and CT-derived JSW should not be interpreted as measurement error alone but rather as reflecting fundamental differences in tissue depiction. Thus, small systematic differences between modalities are expected and should be interpreted in the context of their distinct tissue contrast mechanisms rather than as evidence of measurement inaccuracy. The true hip joint space is anatomically narrow, and cartilage-to-cartilage contact represents the functional joint interface. Therefore, when cartilage is clearly visualized on MERGE, the measured joint space reflects not only osseous spacing but also articular surface integrity, providing clinically meaningful information rather than representing a measurement artifact. Previous radiographic studies have reported that inter-reader variability in hip JSW measurements may reach approximately 1–1.3 mm depending on the measurement method and imaging conditions [16–18]. In this context, Discrepancies of up to approximately ±2 mm are unlikely to substantially alter clinical decision-making in routine DDH assessment, where parameters such as the CE angle, acetabular coverage, and cartilage or labral status typically play a more decisive role. Accordingly, JSW measurements from MERGE should be interpreted as a complementary finding and regarded as a supportive parameter rather than a primary criterion for modality interchangeability.

### Redefining the clinical role of JSW with bone-like MRI

Historically, JSW derived from radiography or CT has served as a surrogate marker of cartilage thickness because cartilage itself could not be visualized. In contrast, MR bone-like imaging enables direct visualization of cartilage, making JSW—when interpreted conventionally—potentially less relevant as a standalone metric. For instance, patients may demonstrate preserved bone-to-bone distance despite focal full-thickness cartilage defects or labral degeneration. Therefore, in the context of MR-based bone-like imaging, JSW should be interpreted as a supportive parameter rather than a definitive marker of joint preservation or degeneration. Accordingly, JSW should not be interpreted in isolation but rather integrated with cartilage status, labral morphology, and acetabular coverage to provide a comprehensive evaluation of joint pathology in DDH.

### CE angle as a stable measurement parameter

**Fig. 5:**
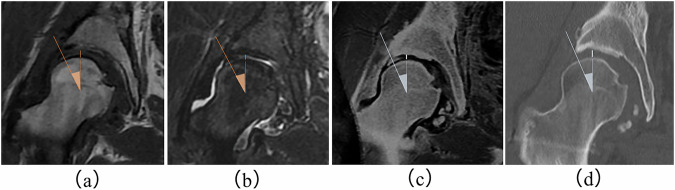
Representative clinical case. A 23-year-old female patient underwent MRI and CT examinations several months after right hip PAO. On coronal images: (**a**) T1WI shows a JSW of 4.9 mm; (**b**) STIR shows 3.6 mm; (**c**) MERGE shows 4.0 mm; and (**d**) CT shows 4.2 mm. The CE angles were: 36° on T1WI, 33° on STIR, 30° on MERGE, and 30° on CT. CT, Computed tomography; MERGE, Multi-echo recombined gradient echo; STIR, Short tau inversion-recovery; T1-WI, T1-weighted imaging

MERGE—after inversion—depicts both cortical bone and articular cartilage with high signal intensity, which may lead observers to place measurement points on the cartilage surface rather than the subchondral bone cortex. As a result, MERGE may underestimate the true osseous JSW compared with CT. Consequently, when using MERGE for MR bone-like imaging of JSW, clinicians should consider the potential influence of cartilage inclusion on the measurements (Fig. 5). In contrast, CE angle measurements were minimally influenced by cartilage depiction, as the lateral acetabular margin represents a well-defined osseous landmark. Unlike the CE angle—which directly reflects acetabular osseous coverage and strongly contributes to diagnostic and surgical decision-making—JSW does not represent a purely anatomical measurement in MRI, where cartilage is visualized. Although MRI sequences such as T1WI and STIR are not cartilage-specific, they can still provide indirect information about cartilage condition through morphology and signal characteristics. Therefore, JSW may serve not as a primary diagnostic indicator but rather as a surrogate parameter relatable to cartilage health, joint preservation planning, or postoperative monitoring. The strong agreement observed in CE angle measurements across all MRI sequences and CT further emphasizes the central role of CE angle as a core radiologic marker, with JSW functioning as a supporting—rather than determinative—metric within clinical workflows.

### Clinical integration and workflow alignment

These results suggest that MERGE can reliably identify bony landmarks, while also offering the advantage of visualizing cartilage and labral structures—allowing a more comprehensive, radiation-free evaluation. In the evaluation of the CE angle, while T1WI and STIR yielded slightly smaller values, MERGE demonstrated an identical mean value (31.4 ± 10.1°) to CT, with no statistically significant differences. This strong agreement can likely be attributed to the anatomical definition of the CE angle, as the measurement point lies at the lateral acetabular margin. At this specific location, the clear MERGE depiction of the bone surface contributes significantly to measurement accuracy (Fig. 5). Furthermore, unlike JSW, CE angle measurements are less affected by cartilage thickness, allowing for a more precise assessment of osseous morphology. These findings indicate that while MERGE provides a reliable visualization of bony landmarks, its performance for CE angle assessment is consistent with that of other MRI sequences. Therefore, multiple MR-based approaches may be suitable for accurate osseous measurements in clinical practice. In our institution, orthopedic surgeons routinely evaluate hip morphology using 2D coronal images, and diagnostic consistency across sequences—including T1WI, STIR, and MERGE—was prioritized. Therefore, the use of 2D MERGE in this study represents not a technical limitation but a clinically aligned design choice. Nevertheless, the present findings may serve as a foundation for future investigations incorporating 3D MERGE or advanced image reconstruction approaches. The 2D acquisition mode employed in this study ensures consistent slice positioning with other routinely acquired 2D sequences, such as T1WI and STIR. This alignment is highly advantageous for clinicians, enabling seamless cross-sectional comparisons and integration of information from various sequences during routine diagnostic evaluations. In busy outpatient settings—where referring physicians often request image comparisons on the same slice as standard MRI sequences (T1WI and STIR)—the use of 2D MERGE proved to be both practical and clinically judicious, facilitating quick and accurate image referencing.

### Advantages, accessibility, and clinical feasibility

The acquisition time for MERGE under the study conditions was approximately 4 min, which is relatively short, allowing easy incorporation into existing clinical MRI protocols. Beyond its technical advantages, the inversion processing of MERGE images can be readily performed on standard medical image viewers, eliminating the need for specialized analysis software or additional equipment. This simplicity results in high reproducibility across clinical sites and low implementation costs, making MERGE a highly practical and accessible method. Moreover, the ability to evaluate cartilage and labral structures supports comprehensive planning for joint-preserving interventions, including conservative therapy and PAO.

### Limitations

This study has several limitations.

First, the sample size was relatively small, comprising 32 hips from patients with DDH. Because the study exclusively focused on DDH and excluded cases with marked femoral head displacement or severe deformity, the findings may be limited to individuals with mild-to-moderate pathology. These factors inherently restrict the generalizability of our results to other hip disorders, such as femoroacetabular impingement, rheumatoid arthritis, or postoperative conditions. In addition, only one hip per patient was included in the analysis, and all observations were treated as independent. Therefore, clustering-related inflation of agreement or reliability estimates was avoided. However, this exploratory study did not formally assess statistical equivalence between MERGE and CT. Future confirmatory studies incorporating equivalence testing methods such as the two one-sided tests procedure will be required.

Second, this study was retrospective and conducted at a single institution. Retrospective data collection may introduce selection bias and variability in imaging timing, while the single-center design limits external validity due to variations in imaging protocols, patient selection, and equipment availability across institutions. Dynamic or functional aspects of hip morphology, such as weight-bearing effects, were also not evaluated.

Third, limitations related to measurement scope and geometric assumptions should be acknowledged. JSW was measured only along the 0° direction on coronal images, which reflects routine clinical practice but does not capture potential regional variations in JSW. In addition, acetabular morphology was evaluated solely using the CE angle, which primarily represents lateral acetabular coverage and does not fully characterize three-dimensional acetabular geometry, including anterior and posterior coverage. Furthermore, pelvic tilt and rotational alignment were not explicitly controlled, and direct image registration between MRI and CT was not performed. These factors may introduce systematic positional or angular measurement bias and should be addressed in future studies incorporating three-dimensional analysis, pelvic alignment correction, and image registration techniques. Therefore, the present findings should be interpreted within the context of an exploratory feasibility study and do not support complete interchangeability between MERGE and CT for JSW assessment. Future studies incorporating three-dimensional reconstruction, geometric correction for pelvic tilt and rotation, and direct image registration between MRI and CT will be required to establish more robust and anatomically standardized measurement frameworks.

Fourth, imaging was performed using a single 1.5-T MRI scanner with only 2D MERGE acquisition available during the study period. The system did not support 3D MERGE, and additional imaging after scanner replacement was not possible. Although the use of 2D MERGE reflected real-world clinical workflow in our institution, the inability to evaluate higher field strengths or 3D acquisitions limits the assessment of MERGE performance across different technical conditions.

Fifth, CT was used as the reference standard for comparison; however, this approach has inherent limitations due to the lack of an independent ground truth. Because CT and MERGE differ fundamentally in tissue depiction—particularly regarding cartilage visualization—systematic modality-dependent differences may influence the observed agreement between measurements. Although measurements were performed independently by two blinded observers and demonstrated excellent reproducibility, some degree of bias cannot be fully excluded. Finally, patient background factors such as body mass index, physique, and bone mineral density were not included in the analysis. These variables may influence bone depiction and MRI signal characteristics and should be incorporated in future investigations.

### Future directions

This study primarily focused on assessing consistency with CT; however, future research should explore the potential of 3D MERGE and the use of artificial intelligence for automated bone structure extraction and analysis [5, 7]. Particularly in cases where CT is contraindicated or undesirable—such as in young patients and those of childbearing age requiring minimal radiation exposure—comprehensive MRI evaluation encompassing both bony and soft tissue structures holds substantial clinical value [1]. The unique ability of MERGE to simultaneously depict cartilage, labrum, and bone structures demonstrates its strong potential as a less invasive yet comprehensive diagnostic modality for a wide range of hip joint disorders. While MERGE demonstrated potential utility for bone morphology assessment, further validation is required before clinical replacement or workflow integration can be recommended. Despite these constraints, the results establish an initial foundation supporting the concept of MR-based bone-like imaging for DDH assessment. Future studies with larger multi-center cohorts, inclusion of multiple MRI platforms, prospective designs, and comparative evaluation of 3D MERGE and advanced artificial intelligence-based reconstruction will be essential to determine its reproducibility, generalizability, and true clinical applicability [8].

## Conclusions

In conclusion, grayscale-inverted MERGE images enable reliable visualization of acetabular morphology and provide quantitative measurements of JSW and CE angle that show good agreement with CT in patients with DDH. In addition to osseous assessment, MERGE simultaneously depicts cartilage and labral structures, allowing a more comprehensive, radiation-free evaluation of the hip joint within routine clinical MRI protocols.

These characteristics suggest that MERGE-based MR bone-like imaging may represent a clinically feasible imaging option for preoperative assessment, particularly in young patients with DDH or in situations where radiation exposure should be minimized. However, this study was exploratory in nature, and MERGE should currently be regarded as a complementary technique to CT. Importantly, in DDH, labral abnormalities and early chondral changes may precede measurable alterations in osseous alignment or joint space narrowing. Therefore, the ability of MERGE to visualize bone morphology together with cartilage and labral pathology enhances its potential clinical value.

### Use of large language models (LLMs)

A large language model (ChatGPT, OpenAI) was used only to refine the English grammar and phrasing during manuscript preparation. No part of the scientific content—including study design, data analysis, interpretation, and figure or table creation—was generated or modified using AI tools. All content produced with AI assistance was thoroughly reviewed and verified by the authors, who retain full responsibility for the accuracy and integrity of the manuscript.

## Abbreviations

3D: Three-dimensional
2D: Two-dimensional
CE: Center-edge
CT: Computed tomography
DDH: Developmental dysplasia of the hip
DICOM: Digital Imaging and Communications in Medicine
ICC: Intraclass correlation coefficient
JSW: Joint space width
MDC95: 95% minimal detectable change
MERGE: Multi-echo recombined gradient echo
MRI: Magnetic resonance imaging
PAO: Periacetabular osteotomy
SEM: Standard error of measurement
STIR: Short tau inversion-recovery
T1WI: T1-weighted imaging
